# Randomized study exploring the combination of radiotherapy with two types of acupuncture treatment (ROSETTA): study protocol for a randomized controlled trial

**DOI:** 10.1186/s13063-017-2139-5

**Published:** 2017-08-29

**Authors:** Rebecca Asadpour, Kerstin A. Kessel, Tom Bruckner, Serkan Sertel, Stephanie E. Combs

**Affiliations:** 1 Department of Radiation Oncology, Technische Universität München (TUM), Klinikum rechts der Isar, Ismaninger Straße 22, 81675 Munich, Germany; 2 Department of Radiation Sciences (DRS), Institute of Innovative Radiotherapy (iRT), Helmholtz Zentrum München, Ingolstädter Landstraße 1, 85764 Oberschleißheim, Germany; 3 Department of Medical Biometry, Institute of Medical Biometry and Informatics (IMBI), Universität Heidelberg, Im Neuenheimer Feld 305, 69120 Heidelberg, Germany; 4 Praxisgemeinschaft Prof. Sertel & Dr. Passerino , Rottstrasse 39, 67061 Ludwigshafen am Rhein, Germany; 5 Deutsches Konsortium für Translationale Krebsforschung (DKTK), Partner Site Munich, Munich, Germany; 6 Department of Otorhinolaryngology, Head & Neck Surgery, University of Heidelberg, Im Neuenheimer Feld 400, 69120 Heidelberg, Germany; 7 Department of 325 Otorhinolaryngology, Head and Neck Surgery, University Hospital CHUV, 326 Bâtiment hospitalier, Rue du Bugnon 46, 1011, Lausanne, Switzerland

**Keywords:** Acupuncture, Radiotherapy, Fatigue, Quality of life (QOL), Sham-controlled trial

## Abstract

**Background:**

Adverse effects such as fatigue, pain, erythema, nausea and vomiting are commonly known in patients undergoing irradiation (RT) alone or in combination with chemotherapy (RCHT). Patients suffering from these symptoms are limited in their daily life and their quality of life (QOL) is often reduced. As addressed in several trials, acupuncture can cause amelioration of these specific disorders. Especially for pain symptoms, several groups have shown efficacy of acupuncture. To what extent the difference between traditional acupuncture (*verum* acupuncture) and false acupuncture (*sham* acupuncture) is in reducing side effects and improvement of QOL is not clear.

**Methods/design:**

ROSETTA is a prospective randomized phase II trial (version 1.0) to examine the efficacy of traditional acupuncture in patients with RT-related side effects. In the experimental (*verum*) arm (n = 37) an experienced acupuncture-trained person will treat dedicated acupuncture points. In the control (*sham*) arm (n = 37) sham acupuncture will be performed to provide a blinded comparison of results.

**Discussion:**

This is the first randomized prospective trial to evaluate the effect of traditional acupuncture on RT-related side effects such as fatigue and QOL.

**Trial registration:**

ClinicalTrials.gov, NCT02674646. Registered on 8 December 2015.

**Electronic supplementary material:**

The online version of this article (doi:10.1186/s13063-017-2139-5) contains supplementary material, which is available to authorized users.

## Background

Radiation therapy (RT) is a main pillar in cancer care offering curative treatment in several indications. However, RT-related side effects develop depending on the site treated (e.g. head and neck or prostate etc.), and on individual patients’ predisposition or other factors. One of the main symptoms related to cancer treatment is the development of fatigue, which can evolve during RT and commonly persists some time after completion of treatment. Other side effects, depending on the region treated, include headache, nausea/vomiting, gastrointestinal discomfort, dysuria and others. Fatigue and subsequently a reduction in overall quality of life (QOL) is a main discomforting factor for patients treated with RT; in many patients fatigue is the only side effect observed during RT. Commonly, this leads to a reduction of daily activity and motivation, and can result in severe unhappiness and/or depression. Thus, methods for amelioration of symptoms and especially any possibilities to reduce fatigue and increase QOL are important factors for patients in the oncology setting.

Several means of supportive care to ameliorate patients’ RT-related side effects are described. In general, it has been shown that moderate exercise can improve QOL and reduce fatigue during and after treatment; additionally, light to moderate exercise can lead to improvement in oncological outcome [1, 2]. Other complementary treatments including selenium, mistletoe or several vitamins are controversial due to limited data on significant benefit; depending on the substances’ mechanisms of action, the risk of RT-related side effects may be increased. Complementary medicine such as traditional Chinese medicine (TCM) or other naturopathy concepts are consistently in discussion and are offered by many practitioners on a case-by-case basis.

For acupuncture, which is on pillar amongst others within the TCM concept, there are some data from randomized studies on back pain, headaches and other non-cancer diseases [3–15]. A large German research consortium, the GERAC-Group, has shown that acupuncture can have a significantly beneficial effect on chronic headache, back pain and joint and bone pain due to arthrosis [16–21]. In oncology, some centers have evaluated the effect of acupuncture on RT-related side effects, such as mucositis and dysphagia, and demonstrated significant reduction of symptoms [3, 10, 22–24]. However, most studies included only a small number of patients, and have not evaluated effects on general fatigue, QOL or other RT-related symptoms.

The prospective phase II ROSETTA trial (version 1.0) will include patients with tumors treated with RT in various anatomical regions. In the experimental arm an experienced acupuncture-trained person will treat dedicated traditional acupuncture points. In the control arm control acupuncture will be performed to provide a blinded comparison of results. Sham acupuncture was developed especially for randomized controlled trials as a valid comparator; compared to a pure control arm, patients receive acupuncture with needlepoints, that have no medical function and thus do not have any proven effect on the symptoms addressed [23–29].

## Methods

### Endpoints of the study

In the ROSETTA trial the main endpoint is improvement of QOL and reduction of fatigue. Secondary endpoints are reduction of RT-related side effects such as headache, nausea and skin erythema.

### Study design

The ROSETTA trial will evaluate the effect of acupuncture as a complementary treatment parallel to RT. Acupuncture is an established and accepted treatment method, which is indicated especially for pain reduction. The plan for study is to recruit 74 patients. The total study duration is planned to be 24 months.

After inclusion into the study and baseline examination, the patient is randomized into the treatment arm. Randomization is performed under the auspices of the biometrician. The randomization process will be performed with the professional web-based patient randomization service, e.g. at www.randomizer.at. The investigator or an authorized member of the study team will perform data documentation and evaluation.

Depending on the treatment arm either *verum* or *sham* acupuncture will be applied [23, 24, 26, 28, 30, 31]. Acupuncture will be performed after treatment bi-weekly during the first week of RT, thereafter weekly until the end of RT. Treatment time in both arms is identical and lies between 20 and 30 minutes. Every patient will be treated according to the treatment arm. Standardized acupuncture needles (sterile needles, e.g. 0.24 × 40 mm) will be used for both treatment types. *Verum* acupuncture is based on foraminology, the ancient Chinese knowledge of acupuncture points [32]. Control acupuncture is performed on unspecific points with no specific effect according to foraminology.

**Fig. 1:**
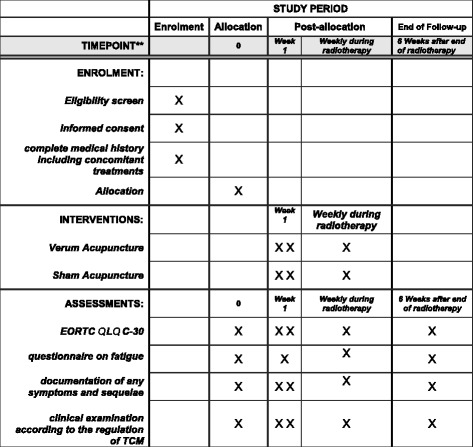
Schedule of enrolment, interventions and assessments in the ROSETTA trial. *EORTC* European Organization for Research and Treatment of Cancer, *QOL* quality of life, *TCM* Traditional Chinese Medicine

Patients will receive standardized questionnaires (European Organization for Research and Treatment of Cancer (EORTC) QLQ C-30) before their first, after their fourth and after their last acupuncture treatment. Patients will be questioned by the investigator about their feelings and symptoms and detailed information about their illness. The answers will be documented according to the standardized scoring system (Common Toxicity Criteria for Adverse Events (CTCAE)). The schedule of enrollment, interventions and assessments in the ROSETTA trial is depicted in Fig. 1.

### Group A: *verum* acupuncture

Needlepoints

Bilateral PC 6, S 36, L 8, L 9

Unilateral R 4, R 6

### Group B: control acupuncture

Needlepoints

Four needles in the medio-axillary line below the 6th rib, bilateral

Two needles unilateral

### Baseline examination

After informed consent and inclusion into the study protocol, a baseline examination will be performed. This examination is to be performed within 2 days before initiation of study treatment and includes:

- complete medical history including concomitant treatments
- clinical examination according to the regulation of TCM
- ascertainment of QOL
- questionnaire on fatigue
- documentation of any symptoms and sequelae prior to radiotherapy

### Follow-up examinations during study treatment

After every acupuncture treatment, an examination will be performed. This examination includes:

- clinical examination according to the regulation of TCM
- ascertainment of QOL
- questionnaire on fatigue
- documentation of any symptoms and sequelae

### Follow up

The final study visit will be 6 weeks after treatment to assess the primary endpoints. After the first 6 weeks, patients will be included into a regular follow-up schedule according to their primary disease and follow-up requirements.

### General criteria for patient selection

All patients treated with RT or radiochemotherapy (RCHT) can be included in the trial if fulfilling the inclusion and exclusion criteria.

### Inclusion criteria

Patients meeting all of the following criteria will be considered for the trial:

- treatment with radiotherapy
- age ≥ 18 years
- ability of subject to understand the nature and individual consequences of the clinical trial
- written informed consent (must be available before enrolment in the trial)

### Exclusion criteria

Patients presenting with any of the following criteria will not be included in the trial:

- any contraindication to acupuncture
- known coagulopathy or anticoagulation therapy with bleeding time > 4 minutes, thrombocyte count < 50 000/μl
- missing compliance
- skin disease in the region of the acupuncture points
- refusal of the patient to take part in the study
- participation in another clinical study or observation period of competing trials

### Statistical considerations

The study is powered to show that continuous acupuncture during RT can have a positive effect on QOL. ROSETTA is a prospective randomized trial comparing traditional (*verum*) acupuncture to control (*sham*) acupuncture. Randomization will be performed using automated information technology (IT)-based randomization, and patients will be allocated to groups by the Study Center within the Department of Radiation Oncology, Technische Universität München (TUM). Additionally, the study will evaluate whether acupuncture can reduce QOL-related side effects such as fatigue, headache, nausea etc.

All patient data on symptoms, side effects and QOL according to the QOL questionnaires will be documented in our dedicated study database. Evaluation of the primary endpoint (QOL) will be performed using the Wilcoxon test (*U* test) and the evaluation tool provided by the EORTC. The secondary endpoints (fatigue, headache, nausea etc.) will be evaluated using comparable statistical calculations. The interpretation of the data will be purely descriptive.

The sample size calculation is based on the Wilcoxon test and the primary endpoint. A sample size of 37 patients per group (i.e. 74 patients in total) is associated with power of 80% to detect a difference between the two groups (with two-sided α = 5%) using the Wilcoxon test, with a probability of 69% that an increase in QOL can be achieved when comparing the two groups 8 (d.h. P(x < y).

### Data management

In the ROSETTA trial the EORTC QLQ-C30 will be used to document the QOL of patients during treatment. Data on side effects and symptoms during RT will be documented on dedicated case report forms (CRFs) designed for the trial and documented in the institutional database. All interventions and every other medical treatment will be recorded. The data will be collected in accordance with all Data Protection Regulations in the MiRO Database of the Department of Radiation Oncology, TUM.

## Discussion

Several studies have shown that traditional acupuncture can alleviate clinical symptoms: In Germany, especially for back pain, a multicenter study group has shown solid data in this regard and provided a strong basis for the use of acupuncture in this regard [16–21].

In oncology, a main focus of acupuncture treatment centers is to reduce RT-related side effects of either chemotherapy or RT. Meng et al. showed that acupuncture can prevent RT-related xerostomia in patients with nasopharyngeal cancer [7, 8]; Lu et al. focused on dysphagia in patients treated with RCHT for tumors of the head and neck, and observed a substantial effect of acupuncture [33]. A comprehensive review on acupuncture in cancer treatment illustrated that there have been 41 randomized controlled trials involving eight different symptoms, such as pain, nausea, hot flashes, fatigue, xerostomia, prolonged postoperative ileus, anxiety/mood disorders and sleep disturbance [34]. Cochrane criteria were used to classify the studies for risk of bias. Only a few studies were identified to be free of risk of bias, or to have a low risk of bias. Thus, more trials focusing on clear questions and applying valid control arms are needed to further clarify the role of acupuncture in oncology care.

To address these questions, the ROSETTA trial was set up as a prospective randomized controlled trial. In the control arm, sham-acupuncture points not associated with any function in the doctrine of TCM are applied (*sham* acupuncture). This concept has been applied in different trials, in patients with and without cancer [3, 7, 8, 23, 24, 33, 35, 36]. Compared to a pure non-treatment controlled trial, the bias of no treatment is not included in this trial concept. In the experimental arm, valid acupuncture points are used, which are known to reduce the associated side effects (*verum* acupuncture). Therefore, the ROSETTA trial will help to clarify the assumed benefit of acupuncture in terms of fatigue and QOL in the oncology setting in patients treated with RT Additional file 1.

### Status of the trial

Recruitment into the ROSETTA trial is ongoing at the time of submission.

## Additional file

Additional file 1: SPIRIT 2013 Checklist: Recommended items to address in a clinical trial protocol and related documents. (DOC 110 kb)

## Abbreviations

CAM: Complementary and alternative medicine
EORTC: European Organization for Research and Treatment of Cancer
QOL: Quality of life
RCHT: Radiochemotherapy
RT: Radiotherapy, radiation therapy
TCM: Traditional Chinese medicine, TUM, Technische Universität München
