# Supplementary material for: Randomized study exploring the combination of radiotherapy with two types of acupuncture treatment (ROSETTA): study protocol for a randomized controlled trial
Source: Trials. 2017 Aug 29;18:398. doi: 10.1186/s13063-017-2139-5 (PMC5575840; doi:10.1186/s13063-017-2139-5)
Supplement: Additional file 1: — SPIRIT 2013 Checklist: Recommended items to address in a clinical trial protocol and related documents. (DOC 110 kb) [file 13063_2017_2139_MOESM1_ESM.doc]

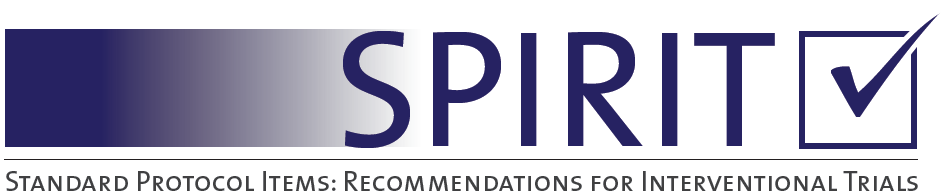


SPIRIT 2013 Checklist: Recommended items to address in a clinical trial protocol and related documents*

| Section/item | ItemNo | Description |
| --- | --- | --- |
| **Administrative information** | | |
| Title | 1 | An adequate title is included:  **R**and**O**mized **S**tudy **E**xploring the combination ofradio**T**herapy with **T**wo types of **A**cupuncture treatment **(ROSETTA):** study protocol for a randomized controlled trial |
| Trial registration | 2a | Clinicaltrials.gov: NCT02674646. Registration Date: 08.12.2015. |
|  | 2b | All relevant information are included in the manuscript. |
| Protocol version | 3 | Version 1.0; 17.11.2015 |
| Funding | 4 | The study is funded in the framework of the Roman Herzog Cancer Center (RHCCC) at the Klinikum rechts der Isar, Onkologisches Zentrum (OZ), Department of Radiation Oncology. |
| Roles and responsibilities | 5a | See page 1 and page 10 |
|  | 5b | See page 1 and page 10 |
|  | 5c | See page 1 and page 10 |
|  | 5d | See page 1 and page 10 |
| Introduction |  |  |
| Background and rationale | 6a | Page 4 and 5 |
|  | 6b | Page 4,5,6 |
| Objectives | 7 | Page 8 |
| Trial design | 8 | Page 7,8 |
| Methods: Participants, interventions, and outcomes | | |
| Study setting | 9 | Page 6-8 |
| Eligibility criteria | 10 | Page 7,8 |
| Interventions | 11a | Page 6 |
|  | 11b | Page 6,7 |
|  | 11c | Page 6,7 |
|  | 11d | N/A |
| Outcomes | 12 | PAGE 5/6 |
| Participant timeline | 13 | PAGE 7/8 |
| Sample size | 14 | PAGE 8 |
| Recruitment | 15 | PAGE 8 |
| **Methods: Assignment of interventions (for controlled trials)** | | |
| Allocation: |  |  |
| Sequence generation | 16a | See page 8 |
| Allocation concealment mechanism | 16b | See page 8 |
| Implementation | 16c | The allocation will be generated by the study center. |
| Blinding (masking) | 17a | n/a |
|  | 17b | n/a |
| **Methods: Data collection, management, and analysis** | | |
| Data collection methods | 18a | Page 8 |
|  | 18b | Page 8 |
| Data management | 19 | Page 8 |
| Statistical methods | 20a | Page 8 |
|  | 20b | n/a |
|  | 20c | Page 8 |
| **Methods: Monitoring** | | |
| Data monitoring | 21a | There is no specific Data monitoring committee. |
|  | 21b | n/a |
| Harms | 22 | n/a |
| Auditing | 23 | N/a |
| Ethics and dissemination | | |
| Research ethics approval | 24 | See page 10 |
| Protocol amendments | 25 | n/a |
| Consent or assent | 26a | See page 10 |
|  | 26b | n/a |
| Confidentiality | 27 | See page 8 |
| Declaration of interests | 28 | Included. No interests. |
| Access to data | 29 | Included into the document. |
| Ancillary and post-trial care | 30 | N/A |
| Dissemination policy | 31a | Data will be published upon completion of the trial. |
|  | 31b | See section participation of co-autors. |
|  | 31c | In principle possible |
| Appendices |  |  |
| Informed consent materials | 32 | AVAILABLE UPON REQUEST |
| Biological specimens | 33 | N/A |

*It is strongly recommended that this checklist be read in conjunction with the SPIRIT 2013 Explanation & Elaboration for important clarification on the items. Amendments to the protocol should be tracked and dated. The SPIRIT checklist is copyrighted by the SPIRIT Group under the Creative Commons “[Attribution-NonCommercial-NoDerivs 3.0 Unported](http://www.creativecommons.org/licenses/by-nc-nd/3.0/)” license.
